# Multimodal dispersal during the range expansion of the tropical house gecko *Hemidactylus mabouia*

**DOI:** 10.1002/ece3.18

**Published:** 2011-10

**Authors:** Kristen H Short, Kenneth Petren

**Affiliations:** Department of Biological Sciences, University of CincinnatiCincinnati, Ohio 45221-0006

**Keywords:** Invasive species, mass dispersal, microsatellite, range expansion

## Abstract

Dispersal influences both the ecological and evolutionary dynamics of range expansion. While some studies have demonstrated a role for human-mediated dispersal during invasion, the genetic effects of such dispersal remain to be understood, particularly in terrestrial range expansions. In this study, we investigated multimodal dispersal during the range expansion of the invasive gecko *Hemidactylus mabouia* in Florida using 12 microsatellite loci. We investigated dispersal patterns at the regional scale (metropolitan areas), statewide scale (state of Florida), and global scale (including samples from the native range). Dispersal was limited at the smallest, regional scale, within metropolitan areas, as reflected by the presence of genetic structure at this scale, which is in agreement with a previous study in this same invasion at even smaller spatial scales. Surprisingly, there was no detectable genetic structure at the intermediate statewide scale, which suggests dispersal is not limited across the state of Florida. There was evidence of genetic differentiation between Florida and other areas where *H. mabouia* occurs, so we concluded that at the largest scale, dispersal was limited. Humans likely contributed to patterns of dispersal at all three scales but in different ways. Infrequent low-volume dispersal has occurred within regions, frequent high-volume dispersal has occurred across the state, and infrequent long-distance dispersal has occurred among continents at the global scale. This study highlights the importance of considering different modes of dispersal at multiple spatial scales to understand the dynamics of invasion and range expansion.

## Introduction

Dispersal plays a critical role in determining the ecological and evolutionary dynamics of range expansion. Natural dispersal in terrestrial habitats has typically been thought to be quite limited and to occur primarily over short distances, giving rise to relatively small founding populations with limited genetic diversity and slow rates of spread (Skellam 1951; Hastings et al. 2005; Hoehn et al. 2007). Human-mediated dispersal, on the other hand, can enhance dispersal by allowing more individuals to disperse over greater distances than would be expected with natural dispersal alone. This additional mode of dispersal can increase founding population size, genetic diversity, dispersal distance, and the rate of spread (Shigesada et al. 1995; Hastings et al. 2005). While human-mediated dispersal has been implicated in the accelerating rates of range expansion in some invasive populations (Andow et al. 1990; Wilson et al. 1999; Suarez et al. 2001), empirical evidence for multiple dispersal modes and their effects on range expansion remains rare. Understanding the role of human-mediated dispersal during range expansion, and particularly during the spread of introduced species, is critical because it can influence both the ecology and evolution of spreading populations.

Human-mediated dispersal can have important demographic and genetic effects, sometimes resulting in high propagule pressure and genetic diversity at the introduction stage of invasion (Kolbe et al. 2004; Dlugosch and Parker 2008). Increased propagule pressure at this stage can prevent invasive populations from experiencing the ecological and genetic effects of small population size (Lockwood et al. 2005; Roman and Darling 2007). In contrast to the large number of studies that have suggested an important role for human-mediated dispersal during introduction, very few studies have investigated the role of humans during subsequent range expansion (Wilson et al. 1999; Suarez et al. 2001; Darling and Folino–Rorem 2009). It cannot be assumed that the role of human-mediated dispersal should be consistent across multiple stages of invasion, because different modes of dispersal may be important for different stages of invasion. For example, while transport on airplanes may be instrumental in introducing individuals to new continents, overland dispersal may be more relevant to their post-introduction spread. Because human-mediated dispersal could accelerate range expansion and thereby increase the extent of impact of invasion on native ecosystems (Parker et al. 1999), it is critical to understand which modes of dispersal are prevalent during spread within the introduced range.

Where multiple modes of dispersal are occurring simultaneously, they are most likely to be detected by investigation of dispersal at multiple spatial scales. Molecular genetic analyses using microsatellites have made it possible to reconstruct dispersal patterns at multiple scales during range expansion (Herborg et al. 2007; Fleischer et al. 2008; Darling and Folino–Rorem 2009). Several genetic analyses of range expansion in aquatic invasive species have found that natural dispersal is most likely to be evident at fine spatial scales, while long-distance human-mediated dispersal is more likely to be evident at larger spatial scales (Wilson et al. 1999; Darling and Folino–Rorem 2009; Dupont et al. 2009). However, few studies have investigated dispersal at multiple spatial scales in terrestrial systems, and this information could be important for managing range expansion, especially in terrestrial urban habitats.

**Figure 1:**
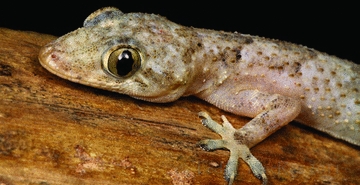
Photograph of *Hemidactylus mabouia*. Like other house geckos, *H. mabouia* typically inhabits building walls in urban areas, but is occasionally found on trees surrounding buildings. It is a nocturnal insectivore, foraging around lights that cluster insects. Photo credit: Ted C. MacRae.

The tropical house gecko *Hemidactylus mabouia* (Fig. 1) is native to Africa and it has been introduced in South America and the Caribbean (Kluge 1969). The species was first recorded on the Florida mainland in 1991 (Butterfield et al. 1993), and has rapidly spread northward throughout the state in the last 20 years (Short and Petren, in review (a)). Dispersal is limited at very small spatial scales (among buildings, tens of meters) in *H. mabouia* (Short and Petren, in review (b)), yet its rapid spread throughout Florida suggests that natural movement is not likely to be the sole means of dispersal of *H. mabouia* at larger spatial scales. Geckos are prime candidates for human-mediated dispersal because of their close habitation with humans, dessication-resistant adhesive eggs with long incubation periods (2 months), and communal nesting (Kluge 1969; Krysko et al. 2003); therefore it is likely that several individuals or eggs can be dispersed rapidly in construction, landscaping, and shipping materials.

**Figure 2:**
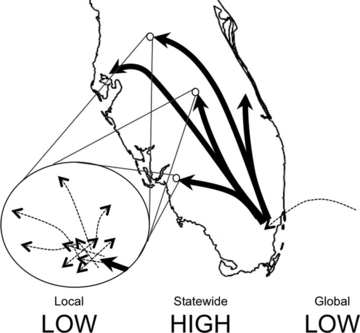
Hypothesized levels of gene flow at three different scales. Gene flow is likely to be low as global colonization events are rare, and dispersal within regions is limited. However, to account for the rapid colonization across Florida, we hypothesize that large numbers of geckos are transported among major metropolitan areas and goods distribution centers across the state.

The goal of this study was to investigate multimodal dispersal in *H. mabouia* during colonization and range expansion in Florida by determining dispersal patterns at multiple spatial scales (Fig. 2). First, we hypothesized that at the regional scale, gene flow is limited, reflecting a combination of natural and low-volume human-mediated dispersal. We therefore predicted we should detect genetic structure among localities within metropolitan regions. Second, we hypothesized that dispersal among regions within Florida is augmented by human transport vectors, and this accounts for the rapid long-distance colonization that has taken place across the state (hundreds of kilometers). We predicted that frequent human transport causes more long-distance dispersal, reduces patterns of isolation by distance, and limits population structure at the larger spatial scales among major cities of Florida. Finally, we hypothesized that at the global scale, transoceanic dispersal is limited, and we therefore predicted that there should be genetic differentiation between Florida and other areas where *H. mabouia* occurs.

## Methods

Gecko tail samples were collected in Florida according to IACUC protocol 06-06-01-01 between May and September in the years 2005–2009. Samples were preserved in 70% EtOH, and microsatellites developed previously in *H. mabouia* were amplified using multiplex PCR (Short and Petren 2008). Fragment analyses were conducted on an AB3730xl DNA analyzer at the Cornell Biotechnology Resource Center and alleles were scored with Genemapper 3.7 (Applied Biosystems, CA, USA).

**Figure 3:**
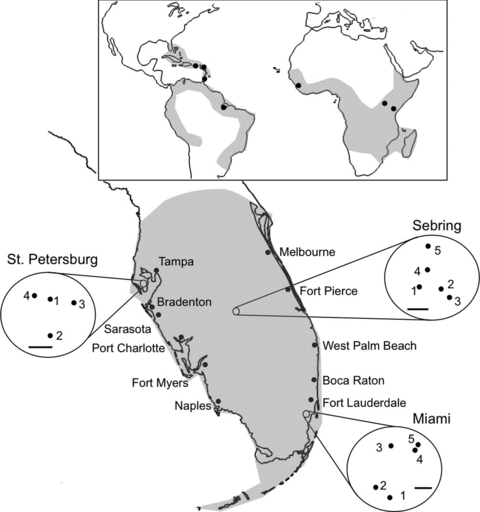
Map showing locations used for sampling. Shaded regions show approximate distribution of *H. mabouia.* Insets show approximate locations of sites used in regional samples, and numbers correspond to those in Table 1. Lines within regional insets represent 10-km scale markers.

We collected 316 samples from building walls at 30 sites in Florida (Table 1). For tests of heterozygosity, allelic richness, and isolation by distance, we excluded the four populations with sample sizes fewer than five individuals to minimize the effects of smaller sample sizes. These populations were included, however in pooled regional analyses of isolation by distance. We also collected nine samples from Maho Bay, St. John, U.S.V.I., and obtained other samples from areas outside Florida from Salvador Carranza (Table 2). We tested for genetic patterns reflecting colonization and gene flow at multiple spatial scales (Fig. 3): the regional scale (multiple sample sites within the same metropolitan area, with maximum distance <60 km), the statewide scale (state of Florida, with maximum distance 300 km), and the global scale (including samples outside Florida).

**Table 1:** Samples used in statewide scale analyses. *N* indicates sample size, *H*_E_ indicates expected heterozygosity, *H*_O_ indicates observed heterozygosity, and *A*_R_ indicates allelic richness. Bold values indicate totals. *H*_O_ values with asterisks indicate significant heterozygote deficits. A cross (X) indicates samples were used only for pooled analyses by region. Numbers in parentheses after site names correspond to regional locations in Fig. 3

	Region	Sample Site	*N*	*H*_E_	*H*_O_	*A*_R_
West Coast	**Naples**	Edison State College, Collier Campus	8	0.50	0.52	2.76
		Laurel Oak Elementary School	12	0.49	0.48	2.74
		**Regional Total**	**20**			
	**Fort Myers**	Canterbury School	13	0.56	0.44*	3.02
		Edison State College, Lee Campus	10	0.51	0.48	2.96
		**Regional Total**	**23**			
	**Port Charlotte**	Port Charlotte High School (X)	3			
		Murdock Middle School (X)	4			
		**Regional Total**	**7**			
	**Sarasota**	Cardinal Mooney High School	15	0.42	0.44	2.27
		**Regional Total**	**15**			
	**Bradenton**	Criminal Justice Academy (X)	2			
		W.D. Sugg Middle School	6	0.48	0.44	2.59
		**Regional Total**	**8**			
	**St. Petersburg**	St. Petersburg College, Clearwater Campus (1)	5	0.53	0.49	2.90
		Fort De Soto Campground (2)	13	0.45	0.38*	2.53
		University of S. Florida, St. Petersburg Campus (3)	10	0.55	0.55	2.93
		Madeira Beach Middle School (X) (4)	3			
		**Regional Total**	**31**			
	**Tampa**	University of South Florida, Tampa Campus	7	0.44	0.40	2.41
		**Regional Total**	**7**			
East Coast	**Miami**	University of Miami (1)	18	0.56	0.49*	2.92
		Florida International University (2)	21	0.55	0.49*	3.05
		St. Thomas University (3)	17	0.58	0.5*	3.09
		Florida International University, Biscayne Bay				
		Campus (4)	8	0.49	0.38*	2.82
		Oleta River State Park (5)	7	0.55	0.56	3.14
		**Regional Total**	**71**			
	**Fort Lauderdale**	University of Florida Agricultural Center	6	0.59	0.54	3.19
		**Regional Total**	**6**			
	**Boca Raton**	Palm Beach State College, Boca Raton Campus	8	0.53	0.54	2.93
		**Regional Total**	**8**			
	**West Palm Beach**	Palm Beach State College, Palm Beach Gardens Campus	5	0.55	0.60	3.12
		**Regional Total**	**5**			
	**Fort Pierce**	Indian River Community College	12	0.56	0.48*	3.06
		University of Florida Agricultural Center	15	0.46	0.41	2.51
		**Regional Total**	**27**			
	**Melbourne**	Florida Institute of Technology	14	0.54	0.49*	2.93
		**Regional Total**	**14**			
Central	**Sebring**	Highlands Hammock State Park (1)	13	0.56	0.53	3.05
		Highlands Regional Medical Center (2)	25	0.52	0.46*	2.94
		Shoppes of the Highlands (3)	21	0.56	0.50	3.25
		Sun'n Lake Elementary (4)	5	0.49	0.58	3.08
		Avon Elementary (5)	10	0.53	0.52	3.19
		**Regional Total**	**74**			

**Table 2:** Samples from three continents. *N* indicates sample size, *H*_E_ indicates expected heterozygosity, *H*_O_ indicates observed heterozygosity, and *A*_R_ indicates allelic richness. Bold values represent totals for the continent

Sample Site	*N*	*H*_E_	*H*_O_	*A*_R_
**Africa**	**5**	**0.64**	**0.47**	**3.44**
Equatorial Guinea	1			
Kenya	2			
Uganda	2			
**South America/Caribbean**	**27**	**0.49**	**0.34**	**2.40**
Brazil	13			
Puerto Rico	1			
Trinidad	2			
Tobago	2			
St. John	9			
**Florida**	**316**	**0.58**	**0.48**	**2.77**
All locations in Table 1				

Observed and expected heterozygosity were calculated in GenAlEx 6.1(Peakall and Smouse 2006), and we used Genepop on the web (Raymond and Rousset 1995) to test for deviations from Hardy–Weinberg equilibrium with a one-tailed test for heterozygote deficit. We corrected for multiple comparisons by adjusting our *P*-values with the sequential Bonferroni correction. We calculated allelic richness in FSTAT v.2.9.3.2 (Goudet 1995) using rarefaction to correct for differences in sample size among sites. We tested for significant differences in allelic richness among sites using Wilcoxon sign-rank tests and alpha of 0.05.

We tested for isolation by distance with Mantel tests conducted in GenAlEx 6.1 (Peakall and Smouse 2006). Geographic distances were obtained using Google Earth, and genetic distances were obtained by estimating *F*_ST_ (θ) in Genetic Data Analysis (GDA; Lewis and Zaykin 2001). For statewide tests, we used approximately linear South–North transects corresponding to the direction of spread and initially considered all populations independently. However, we also pooled populations within the same metropolitan area (e.g., five populations within Miami and five populations within Sebring) to eliminate any potential bias due to genetic differentiation (or lack thereof) at smaller spatial scales. We also conducted an analysis of molecular variance (AMOVA) in GenAlEx to determine the distribution of genetic variation within and among regions in Florida.

We used Bayesian inference in STRUCTURE (Pritchard et al. 2000; Falush et al. 2003; Hubisz et al. 2009) to cluster individuals into populations at both the regional and global scales. In both cases, we conducted simulations with 10,000 iterations of burn-in and 100,000 iterations of Markov Chain Monte Carlo (MCMC), and used the admixture model with correlated allele frequencies and sample location information. For the regional scale, we conducted 10 iterations at each *K* with maximum *K* of 5. For the global scale, we conducted 10 iterations at each *K* with maximum *K* of 3. The most likely value of *K* was determined by plotting mean likelihood at each *K* versus *K*, and determining where the values reached a plateau.

## Results

### Regional scale

**Figure 4:**
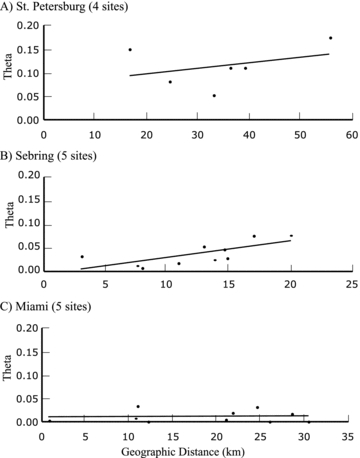
Relationship between geographic distance and genetic distance (*F*_ST_, θ) at the regional scale. A Mantel test produced a significant correlation in the Sebring (B) populations only, but this correlation was not robust to removal of individual populations.

There was no evidence for isolation by distance among four populations in St. Petersburg/Tampa (*r*_M_= 0.35, *P*= 0.35) or five populations in Miami (*r*_M_= 0.055, *P*= 0.47; Fig. 4). There was evidence for isolation by distance among the five populations in Sebring (*r*_M_= 0.72, *P*= 0.02), although the relationship disappeared with removal of the Avon Elementary population (*P*= 0.41), suggesting that this population may have been largely responsible for the correlation.

*F*_ST_ (θ) values among populations within regions ranged from 0 to 0.17 (Table 3). In St. Petersburg/Tampa, the overall θ was 0.13, with 100% of pairwise θ values significant, and in Sebring, the overall θ was 0.039, with 60% of pairwise θ values significant. In both of these regions, θ was significantly greater than zero, indicating the presence of genetic structure. In Miami, however, the overall θ was 0.009, with only 30% of pairwise values significant. This value of θ was not significantly greater than zero and was significantly lower than in both other regions, indicating a relative lack of genetic structure.

**Table 3:** Geographic and genetic distances among multiple sample sites in St. Petersburg, Sebring, and Miami. Geographic distances (km) are below the diagonal and genetic distances (θ) are above the diagonal. Values in bold and with an asterisk indicate statistically significant genetic differentiation according to the Genepop exact test

	St. Petersburg College (SPC)	Fort De Soto	University of S. Florida, St. Petersburg	University of S. Florida, Tampa	
St. Petersburg College (SPC)	–	**0**.110*	**0**.081*	0.053*	
Fort De Soto	36.2	–	**0**.148*	0.173*	
University of S. Florida, St. Petersburg	24.4	16.7	–	0.110*	
University of S. Florida, Tampa	33.1	55.6	39	–	

	**Highlands Hammock**	**Highlands Regional Medical Center**	**Shoppes of Highlands**	**Sun'n Lake Elementary**	**Avon Elementary**
Highlands Hammock	–	**0**.048*	**0**.024*	0.003	0.042*
Highlands Regional Medical Center	13	–	**0**.027*	0.012	0.070*
Shoppes of Highlands	14.8	3.1	–	0.02	0.071*
Sun'n Lake Elementary	8	10.9	13.8	–	0.008
Avon Elementary	14.6	17	20.1	7.6	–

**University of Miami**	**Florida International University**	**St. Thomas University**	**Florida International University, Biscayne**	**Oleta River**	
Universityof Miami	–	0.007	**0**.018*	0.032*	0
Florida International University	10.8	–	0.005	0.017	0
St. Thomas University	21.9	21	–	0.033*	0
Florida International University, Biscayne	24.6	28.6	11	–	0.003
Oleta River	26	30.5	12.2	0.8	–

**Figure 5:**
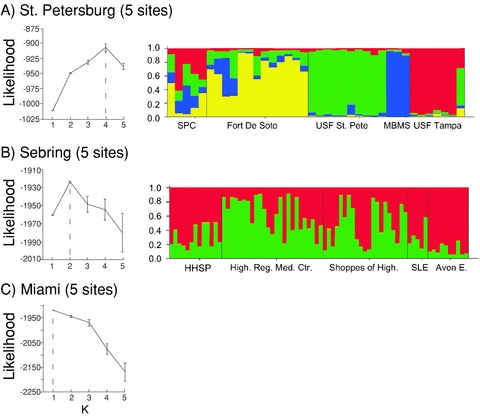
Results of Bayesian clustering analysis in STRUCTURE at the regional scale of analysis. Line graphs show the likelihood of each *K* across 10 runs, and dashed lines indicate the true value of *K*. Bar plots represent clustering patterns in each populations (no plot is given for Miami because no structure was detected). Vertical bars represent individuals, and vertical black lines divide individuals by population. Colors represent proportional membership in each cluster.

In St. Petersburg/Tampa, Bayesian clustering revealed four genetic clusters (*K*= 4) among the five populations. In Sebring, there were two genetic clusters but they did not clearly correspond to sample sites; most sample sites were not strongly assigned to a particular cluster. In Miami, there was only one genetic cluster, indicating relative panmixia at the regional scale (Fig. 5).

An AMOVA on all three regions within Florida revealed that while 0% of the variation was attributable to differentiation among regions, 8% (*P* < 0.001) was attributable to variation within regions. This seemingly counterintuitive result suggested that there was greater structure at a smaller geographic scale than across the state, and was explored further through analyses at the statewide scale.

### Statewide scale

**Figure 6:**
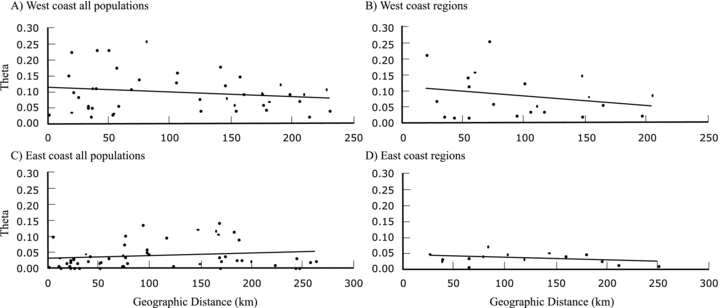
Relationship between geographic distance and genetic distance (θ) in North–South linear transects at the statewide scale. In A and C, all populations were considered independently, while in B and D multiple sample sites within the same region were pooled. No significant correlations were found in any analysis.

Across the state of Florida, expected heterozygosity ranged from 0.42 to 0.59, and allelic richness ranged from 2.27 to 3.19 after rarefaction to four individuals. There was evidence for heterozygote deficit in a few populations (Table 1), which likely reflects substructure within sites (Short and Petren, in review (b)). Despite a wide range of geographic distances among populations, there was no evidence for isolation by distance along the South–North transects on either coast of Florida (Fig. 6). Mantel tests were not significant when populations within regions were considered independently (East coast *r*_M_= 0.16; *P*= 0.20; West coast *r*_M_= 0.18; *P*= 0.09) or when populations within regions were pooled (East coast *r*_M_= 0.29; *P*= 0.20; West coast *r*_M_= 0.057; *P*= 0.17; Fig. 5). There was also no decrease in allelic richness with increasing distance from the most southern population on the East coast (linear regression: *R*^2^= 0.13, *F*_1,9_= 1.37, *P*= 0.27) or the West coast (linear regression: *R*^2^= 0.13, *F*_1,8_= 1.25, *P*= 0.30).

The overall *F*_ST_ (θ) value for all the populations considered in both transects was 0.06. The overall *F*_ST_ (θ) for the West coast transect was 0.11, and 0.07 when populations in the same region were pooled. On the East coast, the overall *F*_ST_ (θ) was 0.04, and 0.03 when regions were pooled. Pairwise θ values among individual populations on either coast ranged from 0 to 0.26 when populations were considered independently and from 0.004 to 0.26 when populations were pooled within regions.

### Global scale

**Figure 7:**
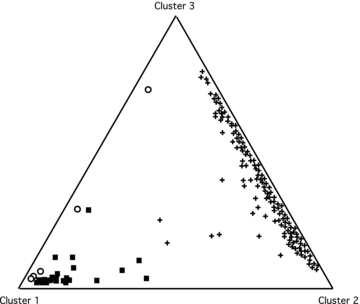
Triangle plot showing results of STRUCTURE analysis. Each point represents an individual from Africa (circles), South America/Caribbean (squares), or Florida (+).

Bayesian population structure analysis revealed the presence of three distinct clusters, with each continent comprising an independent cluster (Fig. 7). When *K*= 2, the S. America/Caribbean samples were clustered with those from Africa, and the Florida samples formed their own cluster. Allelic richness after rarefaction to three individuals (due to few data in the African samples) was significantly different among all three continents, with highest values in Africa, lowest values in South America/Caribbean, and intermediate values in Florida (Africa–S. America/Caribbean *Z*=–34.0; *P*= 0.005; Africa–Florida *Z*=–28.0; *P*= 0.027; S. America/Caribbean–Florida *Z*= 28.0; *P*= 0.027; Table 2). These results suggest that ongoing gene exchange between Florida and these other areas does not play a significant role in determining patterns of genetic structure within Florida.

## Discussion

We tested for genetic structure at three spatial scales, and found different patterns at each scale, which supports our hypothesis of multimodal dispersal (Fig. 2). The presence of genetic structure at the regional scale suggests that gene flow at this scale is limited, and may reflect natural dispersal and/or very limited human-mediated dispersal. This result is congruent with the limited dispersal we have shown at even smaller scales among individual buildings (Short and Petren, in review (b)). Across the state of Florida, however, the lack of pronounced genetic structure suggests that dispersal is not limited and large numbers of individuals are moving greater distances than would be expected with natural dispersal. We conclude that at this scale, human-mediated dispersal is frequent enough to homogenize regions across Florida. At the global scale, we found evidence of genetic structure between Florida and other regions of the world where *H. mabouia* occurs, and this suggests that ongoing dispersal of *H. mabouia* among continents is rare. Together, these results underscore the role of human-mediated dispersal during terrestrial invasion and also demonstrate the importance of investigating patterns at multiple spatial scales during invasion and range expansion.

Significant population structure was present at the regional scale among sites within St. Petersburg and Sebring, although in Sebring, the inferred clusters did not correspond to sample sites. This may reflect recent and ongoing gene flow among sites near Sebring. The lack of genetic structure among sites in Miami is likely attributable to the fact that the Miami area has been colonized by *H. mabouia* for around 20 years. In a separate study, we demonstrate that population structure arising from colonization is ephemeral, and that over time, gene flow among sites gradually erodes the signature of colonization (Short and Petren, in review (b)). When regions are first colonized, individual sites are probably isolated from other nearby sources of migrants, which causes population structure to arise initially. However, over time more neighboring sites become colonized and are able to exchange migrants with previously colonized sites, homogenizing allele frequencies among sites. It is likely that this process explains the lack of genetic structure among Miami populations; this area has been colonized for a long enough period of time to allow gene flow to erase the signature of colonization. The fact that genetic structure arises in the more recently colonized regions suggests, however, that dispersal is limited during range expansion at this scale.

The presence of population structure at the regional scale would appear to contradict the lack of structure at the statewide scale without consideration of multiple dispersal modes. With limited natural dispersal, we would expect to find population structure at both spatial scales. Because we found population structure at the regional scale but not at the larger statewide scale, we conclude that the colonization process must involve different dispersal modes at different spatial scales. In other studies of multimodal dispersal involving natural short-distance dispersal and some form of long-distance dispersal, the long-distance component is generally attributed to either natural long-distance dispersal associated with a particular life stage (especially in aquatic organisms; Kinlan and Gaines 2003), or human-mediated dispersal (Darling and Folino–Rorem 2009). In our gecko system, natural long-distance dispersal seems quite unlikely to occur frequently, because geckos do not have the ability to disperse on wind or water currents, and their natural dispersal is known to be quite limited (Short and Petren, in review (b)). Therefore, human-mediated dispersal is the most likely mechanism by which our results can be explained. Human-mediated transport of geckos seems quite likely to occur, because geckos are known to inhabit and lay eggs on human structures (anecdotal evidence, K. Short pers. obs.).

Even human-mediated dispersal in terrestrial habitats may take different forms at different scales. Dispersal within regions is likely a combination of natural dispersal and low-volume human-mediated dispersal through intentional translocations, localized shipments, or movements on recreational vehicles. However, human-mediated dispersal at the statewide scale is more likely to be a form of mass dispersal through the shipment of larger quantities of goods and containers between hotspot distribution points. Nevertheless, the sharp contrast between the restricted gene flow at the regional scale and the frequent gene flow at the statewide scale suggests that different dispersal modes are prevalent at different spatial scales. In spite of evidence for human-mediated dispersal during the introduction stage of invasion, evidence for this phenomenon during range expansion remains scarce, especially in terrestrial systems. While a few studies have explicitly investigated genetic patterns at multiple scales (Darling and Folino–Rorem 2009; Dupont et al. 2009), ours is unique because it allows us to explore the consequences of range expansion in a terrestrial urban environment.

Many studies have focused on gene flow from the native range as a primary factor in determining the genetic patterns associated with invasion (Dlugosch and Parker 2008). Some studies have found evidence for limited gene flow during introduction (Hawley et al. 2006; Ficetola et al. 2008; Peacock et al. 2009), while a growing number of studies have found that genetic variation is maintained during introduction because of high propagule pressure, multiple introductions, admixture, or rapid population growth (Holland 2001; Zenger et al. 2003; Kolbe et al. 2004; Dlugosch and Parker 2008). In our study, we found some evidence that there was reduced genetic variation in the introduced range compared to the native range, Africa; more importantly, we found evidence of significant genetic structure at this scale. It is perhaps not surprising that there is genetic structure across such large regions of the globe, but for our study it implies that it is unlikely that multiple introductions from genetically distinct sources in Africa occurred as *H. mabouia* colonized the New World, and there is not a significant amount of ongoing gene flow among continents. Therefore, we conclude that transoceanic dispersal in *H. mabouia* is somewhat limited, and does not play a major role in shaping genetic patterns within Florida.

Evidence for scale-dependent human-mediated dispersal has important implications for biological invasions and urban ecology, and these implications extend to a variety of other organisms. The role of human-mediated dispersal in other organisms likely depends to some extent on traits of the organisms. For example, both sociality and commensalism with humans may increase the numbers of individuals involved in human-mediated dispersal events. House geckos are known to share shelters and egg-laying sites on buildings (Krysko et al. 2003), and this makes them ideal candidates for human-mediated dispersal. These features of invading organisms, as well as their prevalence in the pet trade and the extent of intentional introduction by humans, may predispose them to human-mediated dispersal, but landscape features may also be important. It has been suggested that urbanization has led to hyperconnectivity of the landscape for species that inhabit urban areas, because roads and highways connect otherwise fragmented landscapes for such species (Crooks and Suarez 2006). Although roads are barriers to dispersal for many species, they may facilitate mass dispersal in others. Invasive species are particularly likely to be positively influenced by such hyperconnectivity of the environment because they are often closely associated with humans and invade urban habitats (Jeschke and Strayer 2006).

Mass dispersal may have consequences for the ecology and evolution of invasive populations (Wilson et al. 2008). We found evidence for mass dispersal at the statewide scale during range expansion, and the fact that genetic diversity was not lost with successive colonizations suggests that propagule pressure is higher at this scale than it is at the local scale where natural dispersal prevails. Higher propagule pressure suggests that newly colonized populations are probably less affected by drift and Allee effects than populations founded naturally. Human activity increases propagule pressure among regions in the introduced range, and this provides an explanation for how an introduced species, which is capable of only very limited natural dispersal, can spread so rapidly. It also suggests that managing the spread of introduced populations may require consideration of multiple modes of dispersal, especially for species inhabiting urban habitats.

## References

[b1] Andow DA, Kareiva PM, Levin SA, Okubo A (1990). Spread of invading organisms. Landscape Ecol..

[b2] Butterfield BP, Hauge B, Meshaka WE (1993). The occurrence of *Hemidactylus mabouia* on the United States mainland. Herpetol. Rev..

[b3] Crooks JA, Suarez AV, Crooks JA, Sanjayan M (2006). Hyperconnectivity, invasive species, and the breakdown of barriers to dispersal. Connectivity conservation.

[b4] Darling JA, Folino-Rorem NC (2009). Genetic analysis across different spatial scales reveals multiple dispersal mechanisms for the invasive hydrozoan *Cordylophora* in the Great Lakes. Mol. Ecol..

[b5] Dlugosch KM, Parker IM (2008). Founding events in species invasions: genetic variation, adaptive evolution, and the role of multiple introductions. Mol. Ecol..

[b6] Dupont L, Viard F, Dowell MJ, Wood C, Bishop JDD (2009). Fine- and regional-scale genetic structure of the exotic ascidian *Styela clava* (Tunicata) in southwest England, 50 years after its introduction. Mol. Ecol..

[b7] Falush D, Stephens M, Pritchard JK (2003). Inference of population structure using multilocus genotype data: linked loci and correlated allele frequencies. Genetics.

[b8] Ficetola GF, Bonin A, Miaud C (2008). Populations genetics reveals origin and number of founders in a biological invasion. Mol. Ecol..

[b9] Fleischer RC, Boarman WI, Gonzalez EG, Godinez A, Omland KE, Young S, Helgen L, Syed G, McIntosh CE (2008). As the raven flies: using genetic data to infer the history of invasive common raven (*Corvus corax*) populations in the Mojave Desert. Mol. Ecol..

[b10] Goudet J (1995). FSTAT (Version 1.2): a computer program to calculate F-statistics. J. Hered..

[b11] Hastings A, Cuddington K, Davies KF (2005). The spatial spread of invasions: new developments in theory and evidence. Ecol. Lett..

[b12] Hawley DM, Hanley D, Dhondt AA, Lovette IJ (2006). Molecular evidence for a founder effect in invasive house finch (*Carpodacus mexicanus*) populations experiencing an emergent disease epidemic. Mol. Ecol..

[b13] Herborg LM, Weetman D, Van Oosterhout C, Hanfling B (2007). Genetic population structure and contemporary dispersal patterns of a recent European invader, the Chinese mitten crab, *Eriocheir sinensis*. Mol. Ecol..

[b14] Hoehn M, Sarre SD, Henle K (2007). The tales of two geckos: does dispersal prevent extinction in recently fragmented populations. Mol. Ecol..

[b15] Holland BS (2001). Invasion without a bottleneck: microsatellite variation in natural and invasive populations of the brown mussel *Perna perna* (L). Mar. Biotechnol..

[b16] Hubisz M, Falush D, Stephens M, Pritchard JK (2009). Inferring weak population structure with the assistance of sample group information. Mol. Ecol. Res..

[b17] Jeschke JM, Strayer DL (2006). Determinants of vertebrate invasion success in Europe and North America. Glob. Change Biol..

[b18] Kinlan BP, Gaines SD (2003). Propagule dispersal in marine and terrestrial environments: a community perspective. Ecology.

[b19] Kluge AG (1969). The evolution and geographical origin of the New World Hemidactylus mabouiabrookii complex (Gekkonidae, Sauria).

[b20] Kolbe JJ, Glor RE, Schettino LR (2004). Genetic variation increases during biological invasion by a Cuban lizard. Nature.

[b21] Krysko KL, Sheehy CM, Hooper AN (2003). Interspecific communal oviposition and reproduction of four species of lizards (Sauria: Gekkonidae) in the lower Florida Keys. Amphibia-Reptilia.

[b22] Lewis PO, Zaykin D (2001). Genetic data analysis: computer program for the analysis of allelic data. http://hyrodictyon.eeb.uconn.edu/people/plewis/software.php.

[b23] Lockwood JL, Cassey P, Blackburn T (2005). The role of propagule pressure in explaining species invasions. Trends Ecol. Evol..

[b24] Parker IM, Simberloff D, Lonsdale WM (1999). Impact: toward a framework for understanding the ecological effects of invaders. Biol. Invasions.

[b25] Peacock MM, Beard KH, O'Neill EM, Kirchoff VS, Peters MB (2009). Strong founder effects and low genetic diversity in introduced populations of Coqui frogs. Mol. Ecol..

[b26] Peakall R, Smouse PE (2006). GENALEX 6: genetic analysis in Excel. Population genetic software for teaching and research. Mol. Ecol. Notes.

[b27] Pritchard JK, Stephens M, Donnelly P (2000). Inference of population structure using multilocus genotype data. Genetics.

[b28] Raymond M, Rousset F (1995). GENEPOP (version 1.2): population genetics software for exact tests and ecumenicism. J. Hered..

[b29] Roman J, Darling JA (2007). Paradox lost: genetic diversity and the success of aquatic invasions. Trends Ecol. Evol..

[b30] Shigesada N, Kawasaki K, Takeda Y (1995). Modeling stratified diffusion in biological invasions. Am. Nat..

[b31] Short KH, Petren K (2008). Isolation and characterization of twelve polymorphic microsatellite markers in the tropical house gecko (*Hemidactylus mabouia*). Mol. Ecol. Res..

[b32] Short KH, Petren K

[b33] Short KH, Petren K

[b34] Skellam JG (1951). Random dispersal in theoretical populations. Biometrika.

[b35] Suarez AV, Holway DA, Case TJ (2001). Patterns of spread in biological invasions dominated by long-distance jump dispersal: insights from Argentine ants. Proc. Nat. Acad. Sci. U.S.A..

[b36] Wilson AB, Naish KA, Boulding EG (1999). Multiple dispersal strategies of the invasive quagga mussel (*Dreissena bugensis*) as revealed by microsatellite analysis. Can. J. Fish. Aquat. Sci..

[b37] Wilson JRU, Dormontt EE, Prentis PJ, Lowe AJ, Richardson DM (2008). Something in the way you move: dispersal pathways affect invasion success. Trends Ecol. Evol..

[b38] Zenger KR, Richardson BJ, Vachot-Griffin A-M (2003). A rapid population expansion retains genetic diversity within European rabbits in Australia. Mol. Ecol..

